# Patient satisfaction survey for HAM-net registrants

**DOI:** 10.1186/1742-4690-12-S1-P40

**Published:** 2015-08-28

**Authors:** Kentaro Sato, Takayuki Kikuchi, Miyuna Kimura, Midori Komita, Kanade Shimada, Krumi Seki, Marika Tachibana, Naoko Yagishita, Ariella Coler-Reilly, Tomoo Sato, Natsumi Arayta, Miho Ishikawa, Mikako Koike, Yumi Saito, Hiroko Suzuki, Ayako Takata, Yoshihisa Yamano

**Affiliations:** 1 St. Marianna University School of Medicine, Kawasaki, Kanagawa, 2168511, Japan; 2 Department of Rare Diseases Research, Institute of Medical Science, St. Marianna University School of Medicine, Kawasaki, Kanagawa, 2168511, Japan; 3 Intractable disease Consultation, St. Marianna University School of Medicine, Kawasaki, Kanagawa, 2168511, Japan; 4 Department of Preventive Medicine, St. Marianna University School of Medicine, Kawasaki, Kanagawa, 2168511, Japan

## 

Patient registries can foster communication between doctors and patients who have rare diseases. In order to succeed in the long term, the managers of the registry must work to raise the satisfaction of the registrants. We conducted a satisfaction survey for the HTLV-1 associated myelopathy (HAM) patient registry known as “HAM-net.” Based on the data we collected, we made several alterations, mainly to the newsletter and website. This year we repeated the survey to determine if our alterations were beneficial or not by assessing changes in satisfaction levels. We surveyed 368 patients registered to HAM-net, inquiring about their general impression of HAM-net, the requisite telephone interviews, the newsletter, and the website. The response rate was 74.7%, and 198 registrants responded to the satisfaction survey both times. The number of individuals satisfied with HAM-net in general improved from 63.6% to 77.3% of registrants. More specifically, satisfaction with the newsletter rose dramatically but no significant change was observed for the website. Therefore we concluded that focusing on the newsletter is currently the best way to improve patient satisfaction. For example, we deduced that it is beneficial to increase the volume and quality of the articles. On the other hand, we could not demonstrate that frequently updating the website was beneficial to the patients. We inferred that the website must be improved to be more appealing and useful to the patients. In this study, we showed that properly investigating patient satisfaction using a detailed questionnaire can produce the data necessary to improve patient satisfaction levels. The best way to preserve a good relationship with the registered patients in the long term is to prioritize understanding the patients’ needs and continuously work to improve their satisfaction.

